# Cyclophilin A interacts with diverse lentiviral capsids

**DOI:** 10.1186/1742-4690-3-70

**Published:** 2006-10-12

**Authors:** Tsai-Yu Lin, Michael Emerman

**Affiliations:** 1 Pathobiology Graduate Program, University of Washington, Seattle, WA 98195, USA; 2 Division of Human Biology, Fred Hutchinson Cancer Research Center, Seattle, WA 98109, USA

## Abstract

**Background:**

The capsid (CA) protein of HIV-1 binds with high affinity to the host protein cyclophilin A (CypA). This binding positively affects some early stage of the viral life-cycle because prevention of binding either by drugs that occupy that active site of cyclophilin A, by mutation in HIV-1 CA, or RNAi that knocks down intracellular CypA level diminishes viral infectivity. The closely related lentivirus, SIVcpz also binds CypA, but it was thought that this interaction was limited to the HIV-1/SIVcpz lineage because other retroviruses failed to interact with CypA in a yeast two-hybrid assay.

**Results:**

We find that diverse lentiviruses, FIV and SIVagmTAN also bind to CypA. Mutagenesis of FIV CA showed that an amino acid that is in a homologous position to the proline at amino acid 90 of HIV-1 CA is essential for FIV interactions with CypA.

**Conclusion:**

These results demonstrate that CypA binding to lentiviruses is more widespread than previously thought and suggest that this interaction is evolutionarily important for lentiviral infection.

## Background

Cyclophilin A (CypA) is a highly conserved peptidyl prolyl isomerase (PPIA) that is incorporated into HIV-1 virions and plays a yet undefined role in the early stages of viral replication in some cell types [1-3]. CypA interacts with HIV-1 by virtue of a direct binding between residues in a loop between the fourth and fifth alpha-helices of the capsid (CA) protein of HIV-1 and the active site of CypA [4-6]. Cyclosporin A (CsA), an immunosuppressive drug, binds to the same region of binding groove of CypA and disrupts the CypA/CA interaction which leads to an attenuation of wild type HIV-1 infectivity by 2–5 fold in T cells [7-9]. Although CypA can bind to viral CA in the producer cell during viral assembly, it is CypA in the newly infected target cells that is important for infectivity rather than the CypA that is present in the producer cell [8-11]

Tripartite motif 5 isoform alpha (Trim5α) proteins also bind to retroviral CA early after viral entry, and can have a negative effect on the viral lifecycle by accelerating the viral core uncoating or CA degradation [12-15]. Trim5α contains a C-terminal B30.2 domain that recognizes retroviral CA and restricts viral replication in a species-specific manner [12,16]. The resistance of cells from the owl monkey (*Aotus trivirgatus*) to HIV-1 infection is due to the presence of a natural fusion protein in this species, called TrimCyp, in which the B30.2 recognition domain of Trim5α was replaced by the *CypA *gene [17,18]. CypA is important for the activities of some Trim5α alleles [19,20].

The CypA/CA interaction was initially identified by two-hybrid analysis in yeast [1,5]. In those studies, it was reported that only HIV-1 and the closely related SIVcpz encoded Gag proteins (which are cleaved by viral protease to generate CA during maturation) that could interact with CypA, while the Gag proteins from HIV-2, SIVmac, SIVs from African green monkeys-Sabaeus (SIVagmSAB), feline immunodeficiency viruses (FIV), and Mason-Pfizer monkey viruses (MPMV) failed to interact with CypA in this assay [5]. Furthermore, incorporation of CypA into virions was detected in HIV-1 and SIVcpz, but not in HIV-2, SIVmac, SIVagm-Grivet (SIVagmGRI), and murine leukemia viruses (MLV) [5]. However, here we show that the CypA/CA interaction is not unique to HIV-1/SIVcpz. By both genetic and biochemical experiments we show that FIV can bind CypA and its replication is affected by this interaction to the same extent as HIV-1. Moreover, we have identified an amino acid that is essential for FIV CA interaction with CypA that is in a nearly identical context to an amino acid necessary for the interaction of HIV-1 CA with CypA. Finally, we show that SIVagm-Tantalus (SIVagmTAN) is restricted by TrimCyp, strongly suggesting that this viral CA also interacts with CypA. While these studies were in progress, two other groups also provided evidence that FIV and SIVagm are both susceptible to the TrimCyp restriction in a CsA sensitive manner [21,22]. Together, these results demonstrate that lentiviral interactions with CypA are more conserved than had been previously assumed and suggest that CypA/CA interactions play an evolutionarily conserved role in the life cycle of many lentiviruses.

## Results

### FIV CA interacts with CypA

We began this study by looking at the pattern of restriction effect of Trim5α isolated from diverse primate species with a panel of different retroviruses. Remarkably, we found that HIV-1 and FIV had an identical pattern of restriction in that both were strongly restricted by rhesus Trim5α, had slight sensitivity to human and Tamarin Trim5α, and were resistant to Titi Trim5α (data not shown). Similar data was also reported by another group [23]. Because Trim5α restriction, like the CypA interaction, is dictated by CA, this led us to examine if FIV is also similar to HIV-1 in its interaction with CypA.

**Figure 1:**
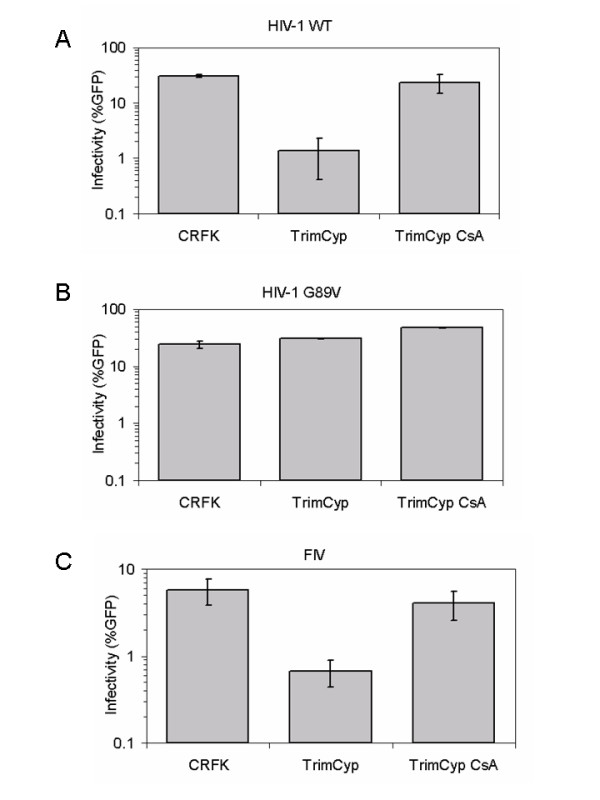
**Infectivity of HIV-1 WT, G89V mutant and FIV under the TrimCyp restriction**. CRFK and CRFK-TrimCyp cells were infected with HIV-1 wild-type (A), HIV-1 G89V (B), and FIV (C) in the presence or absence of CsA. The infected cells were analyzed by flow cytometry and the infectivity is presented as percentage of cells that were GFP+ (on a log scale).

The TrimCyp protein from owl monkeys strongly restricts HIV-1 because the C-terminal CypA portion of the protein binds to HIV-1 CA, while the N-terminal portion of the protein leads to premature uncoating or degradation of incoming virions [13,14,17,18]. We therefore asked whether FIV is also sensitive to TrimCyp restriction as an indirect measure of CA-CypA recognition. Cells expressing TrimCyp were generated and infected with VSV-G pseudotyped wild-type HIV-1, a HIV-1 G89V mutant (CA with a G89V mutation does not bind CypA [2]), and FIV (Fig. 1). Infections were done in the presence or absence of CsA to verify the dependence of the restriction on CypA function. Because infections were done with viruses that encode GFP (see Methods), the number of infected cells could be directly analyzed by flow cytometry (Fig. 1). Consistent with previous reports [17,18], HIV-1 is sensitive to TrimCyp restriction and the restriction can be neutralized by treatment of cells with CsA (Fig. 1A), while the negative control, the HIV-1 G89V mutant, is not restricted by TrimCyp (Fig. 1B). We found that FIV behaves similar to HIV-1 in that it was restricted by TrimCyp, and this restriction is reversed by CsA (Fig. 1C).

**Figure 2:**
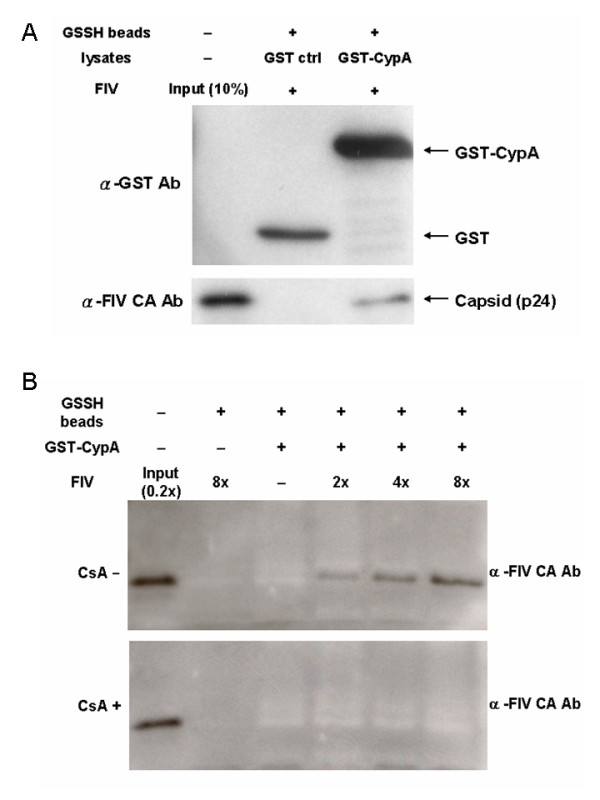
**Biochemical interaction of CypA and FIV CA**. (A) GST-CypA or control GST fusion proteins were generated in 293T cells. After the incubation of the 293T cell lysates with glutathione beads, the beads were washed to remove unbound proteins, and then incubated with FIV virions. After removing unbound FIV virions by washing, beads were subjected to the SDS-PAGE and probed with anti-FIV CA antibodies for the detection of the interaction between CypA and FIV CA. (B) was conducted with the same experimental protocol for (A), but with different amounts of input FIV virions (serial dilution) in the presence or absence of 10 μM of CsA.

These results with TrimCyp suggested that FIV does indeed interact with CypA. In order to test this directly, we used an assay to biochemically detect binding of CypA to FIV CA (Fig. 2). Thus, 293T cells were transiently transfected with a plasmid that expressed a fusion protein between GST and human CypA (GST-CypA) or with GST alone. Cell lysates were then incubated with glutathione beads to partially purify the GST proteins from the cell extracts. After washing to remove unbound proteins, the beads were incubated with FIV virions, and the GST-bound proteins were run on SDS-PAGE gels and blotted with anti-CA antibodies. HIV-1 WT and G89V were used as the positive and negative controls for this assay, respectively. HIV-1 wild type associates with CypA because it is pulled down by the GST-CypA fusion protein, whereas the HIV-1 G89V mutant does not bind to GST-CypA (data not shown). On the other hand, FIV behaves similar to HIV in that partially purified GST-CypA, but not GST alone, is able to bind FIV *in vitro *(Fig. 2A). This binding is dose-dependent (adding more FIV virions increases the amount of CA that is pulled-down, Fig. 2B, top), and is sensitive to CsA (Fig. 2B, bottom). Thus, by both genetic (TrimCyp restriction) and biochemical (GST-CypA pull-downs) assays, we demonstrate that FIV, like HIV-1, interacts with CypA.

### Proline 90 on FIV capsid is an essential amino acid for Cyclophilin A binding

**Figure 3:**
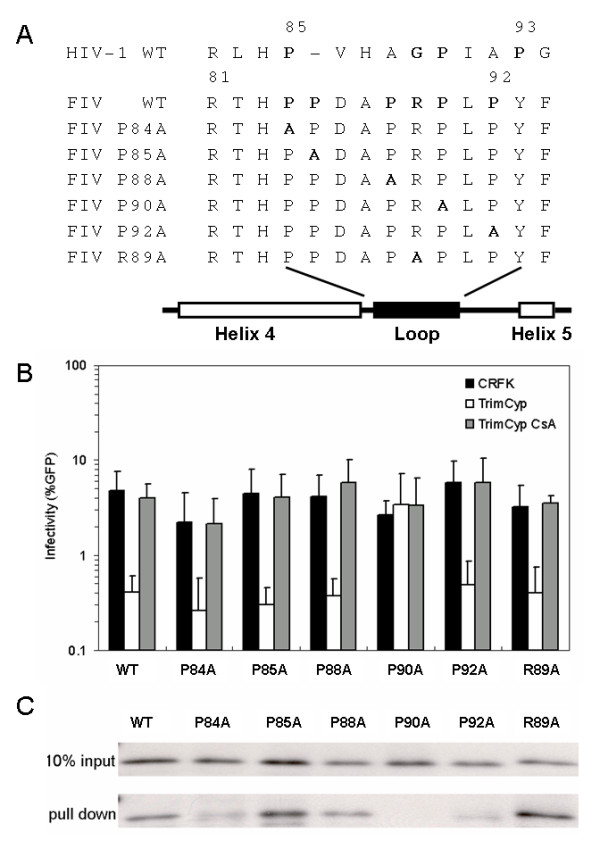
**CypA/CA interaction in FIV WT and mutants**. (A) The CA amino acid sequence alignment of FIV with HIV-1. The proline to alanine and the arginine to alanine mutations are in bold, and gaps are indicated with "-". (B) Infectivity of FIV wild type and mutants under the TrimCyp restriction. FIVs wild type and mutants were transduced to CRFK or CRFK-TrimCyp cells to test their sensitivity to TrimCyp restriction. The % GFP determined by flow cytometry is the readout of the infectivity. (C) GST-CypA pull-down of CA from wild type and mutant FIV. The upper panel shows 10% of input FIV virions used for the GST-CypA pull-down assay, and the lower panel shows the CA pulled down by GST-CypA. FIV virions are detected by anti-FIV CA antibody.

The glycine at position 89 and the proline at position 90 on HIV-1 capsid are the most critical target amino acids of CypA binding [2]. Since we observed that FIV also interacts with CypA, we next tried to identify if amino acids in a similar region of CA of FIV are also critical for the CypA recognition. By aligning the capsid amino acid sequence of FIV with that of HIV-1, we noticed that FIV capsid contains five prolines on the loop between the predicted alpha-helices 4 and 5 (Fig. 3A). Each of these five proline residues was individually mutated to alanine. In addition, arginine at position 89 was also mutated to alanine because this position corresponds to the critical glycine at position 89 in the HIV CA (Fig. 3A).

These constructs were then co-transfected to 293T cells with VSV-G and GFP reporter plasmids to generate VSV-G pseudotyped FIVs that were used to infect CRFK or CRFK-TrimCyp cells. The infectivity of each virus was normalized to give an amount of virus that resulted in about the same number of infected CRFK cells. Similar to wild-type FIV, the mutations P84A, P85A, P88A, P92A, and R89A are also restricted by TrimCyp (Fig. 3B). Moreover, in each case, the restriction by TrimCyp is abrogated by the addition of CsA. These data indicate that mutating each of these residues did not destroy a functional interaction between CypA and the viral CA. However, the P90A mutant is not sensitive to the TrimCyp restriction and the infectivity is not affected by the addition of CsA. This indicates that amino acid P90 is critical for a functional interaction between FIV CA and CypA (Fig. 3B).

In addition to the TrimCyp assay, we also applied the GST-CypA pull down assay to test the interaction of CypA and FIV mutants. The FIV wild type and mutant virions were incubated with the GST-CypA and the interactions were analyzed by Western Blotting (Fig. 3C). Wild-type FIV and other mutants are sensitive to the TrimCyp restriction and are pulled down by the GST-CypA. On the other hand, the P90A mutant, which is not sensitive to the TrimCyp restriction, is also not pulled down by GST-CypA (Fig. 3C). These data demonstrate that amino acid P90 is a critical target for CypA binding to FIV CA.

### CypA affects FIV infection

**Figure 4:**
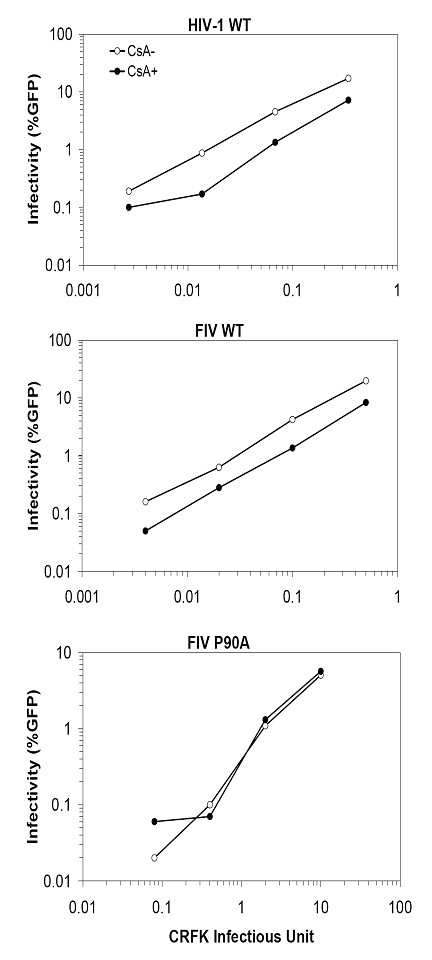
**The CypA-dependency of FIV**. Jurkat T cells were infected with HIV-1 wild-type (A), FIV wild-type (B), and FIV P90A (C) in the presence or absence of 1.25 μM of CsA. All viruses were first normalized on CRFK cells and equivalent CRFK infectious units were used and are plotted on the X-axis. For example, the amount of virus that gave 20% GFP positive cells on CRFK cells is 0.2 on the X-axis. The infected Jurkat cells were analyzed by flow cytometry and the infectivity is presented as % GFP+ cells.

Previous reports showed that CsA decreases spreading infections of FIV in cells and in animals [24,25]. However, comparisons with HIV-1 were not done. It has been shown that the infectivity of HIV-1 drops 2–5 fold when the CypA/CA interaction is blocked by CsA in Jurkat T cells in single-cycle infection experiments [7-9]. To examine the role of CypA in FIV infection in the same cell type, Jurkat T cells were infected with VSV-G-pseudotyped FIV in the presence or absence of CsA (Fig. 4). As expected, the infectivity of wild type HIV-1 was reduced by the addition of CsA (Fig. 4, top). We obtained reduced infections of FIV in Jurkat cells relative to CRFK cells presumably due to human Trim5α restriction of FIV [23]. However, similar to HIV-1, the infectivity of FIV decreased further by 2–5 fold when CsA was present during infection (Fig. 4, middle). The P90A mutant of FIV which fails to bind cyclophilin A (Fig. 3), in contrast, was not sensitive to CsA treatment (Fig. 4, bottom). These results indicate that FIV, like HIV-1, requires the endogenous cyclophilin A in target cells for optimal infection.

### SIVagmTAN also binds to CypA

**Figure 5:**
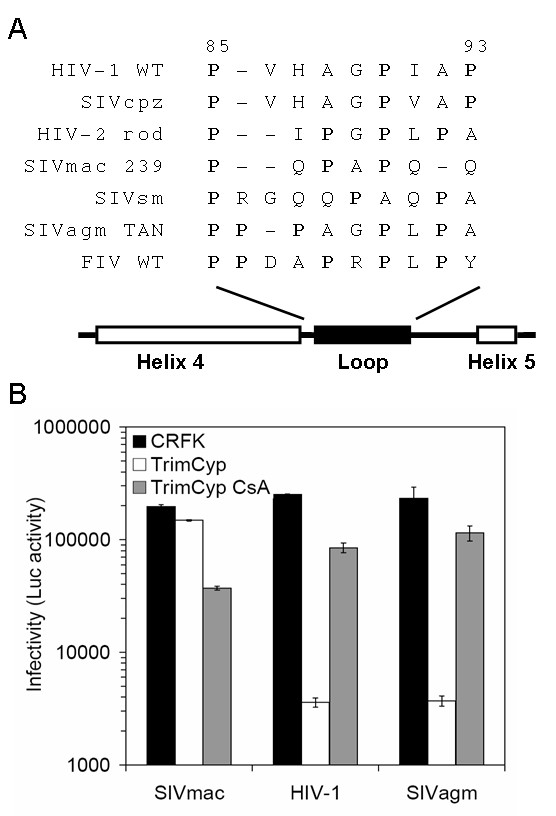
**CypA/CA interaction in SIVmac, HIV-1, and SIVagmTAN**. (A) Sequence alignment of HIV-1 and other lentiviruses. The amino acid sequences of the loop between α helices 4 and 5 were aligned with HIV-1. Prolines are in bold, and gaps are indicated as "-". (B) Infectivity of HIV-1, SIVmac, and SIVagmTAN under the TrimCyp restriction. CRFK or CRFK-TrimCyp cells were infected with HIV-1-luc, SIVmac-luc, and SIVagmTan-luc, and the infectivity was determined by luminometer.

We aligned the amino acids in the region between the prediced alpha-helices 4 and 5 of CA from a number of different lentiviruses, and noticed that SIVagmTAN, similar to FIV, has 5 prolines, and that the length of the SIVagmTAN loop (9 amino acids) is the same as HIV-1 (Fig. 5A). We therefore tested whether SIVagmTAN also interacts with CypA using the TrimCyp assay (Fig. 5B). Consistent with other reports [5,6], SIVmac, as a negative control, is not sensitive to the TrimCyp. On the other hand, SIVagmTAN behaves like HIV-1 and FIV. The infectivity of SIVagmTAN is strongly restricted by TrimCyp protein, and the restriction can be counteracted by the treatment of CsA (Fig. 5B). This result suggests that the CA of SIVagmTAN is also recognized by the CypA, and that the ability of CypA to recognize lentiviral capsids is widespread, although not universal, among lentiviruses.

## Discussion

The CypA/CA interactions had originally been described only for HIV-1 and SIVcpz [5]. Here, we show that this phenotype is more widespread among lentiviruses because both FIV and SIVagmTAN also interact with CypA. Moreover, we identified an amino acid in FIV CA that is critical for CypA binding that is in a similar position in CA of HIV-1. Finally, we show that the CypA/CA interaction is functionally significant for FIV replication.

It is not clear why a functional assay (Fig. 1) and a direct binding assay (Fig. 2) detected the CypA/CA interaction in FIV, whereas previous reports that looked for interactions with a yeast-two hybrid assay or by expression of recombinant Gag and CypA in *E. Coli *did not [5]. It is possible that the formation of mature viral core may be more important for CypA recognition of FIV, or that subtle folding problems with FIV Gag was expressed in yeast and *E. Coli *might have prevented binding. Nonetheless, the two assays used here (TrimCyp restriction and GST-CypA pull down) were conducted in mammalian cells and thus more closely mimic the structure of CA found in natural targets cells.

The finding that FIV and SIVagmTAN also bind host CypA demonstrates that the CypA/CA interaction is a more wide spread phenotype among lentiviruses than just the HIV-1/SIVcpz lineage. At this point it is not possible to determine if this property arose independently in three separate lentivirus lineages, or if an ancestral retrovirus was able to bind CypA and then other lentiviruses (for example, SIVmac) each lost this ability. Although the latter possibility is more parsimonious, it will be important to test a much broader range of lentiviruses for CypA binding. It is also possible that the ability of a lentivirus to bind CypA is evolutionarily dynamic and changes upon adaptation to new hosts.

**Figure 6:**
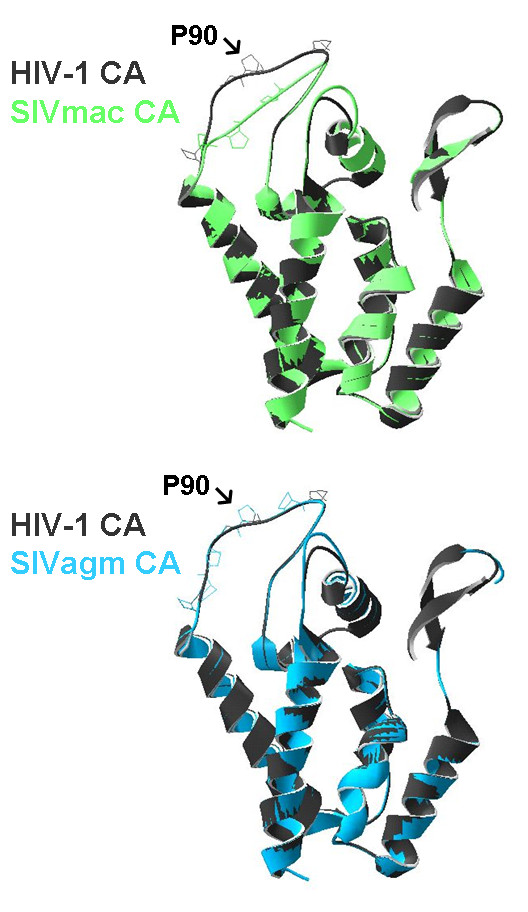
**Computer modelling of the CA structure from SIVmac and SIVagmTAN**. The CA structure from SIVmac and SIVagmTAN by using HIV-1 CA crystal structure (PDB:1AK4) as template. The CA from SIVmac (upper panel) and SIVagmTAN (lower panel) were superimposed on the CA from HIV-1 which binds to CypA. The proline at position 90 on HIV-1 CA is indicated.

We determined that the proline at amino acid 90 on FIV CA is critical for the interaction with CypA. This is similar to the site on HIV-1 that binds CypA, suggesting the proline in the middle of the loop is crucial for CypA recognition. However, the CA sequence alignment of SIVmac with HIV-1 shows that SIVmac also possesses this corresponding proline (Fig. 5A). The major difference of the loop between the fourth and fifth alpha-helices in HIV-1 and SIVmac is its length. We applied the SWISS-MODEL computer modelling to generate putative structure of CA from SIVmac and SIVagmTAN based on the crystal structure of HIV-1 CA bound to CypA (PDB:1AK4). We found that the CypA-binding loop of SIVagmTAN can be superimposed on the parallel loop of HIV-1 well, while the loop of SIVmac is shorter than that of HIV-1 and is not able to fit in the HIV-1 scale (Fig. 6). This suggests that in addition to the proline at position 90 (based on HIV-1), the length of the loop also contributes to the CypA binding. A shorter loop might not be able to insert into the binding groove of CypA even though the target proline is present in the middle of the loop. On the other hand, Cyclophilin B (CypB), another member in the cyclophilin family, was showed to interact with Gag proteins from HIV-1 and SIVmac [1,2]. However, the CypB/Gag interaction in both HIV-1 and SIVmac is not mediated by binding of CypB to the same loop that CypA recognizes. To our knowledge, the CypB/Gag interaction has not been reported to affect viral replication.

The exact role of CypA/CA interaction in HIV-1 life cycle is still not clear. However, several groups have suggested that CypA/CA interaction mediates the lentiviral susceptibility to Trim5α restriction [17-19,26,27]. The discovery of TrimCyp links the CypA/CA interaction with the post-entry restriction mechanism [17,18]. The potent restriction against HIV-1 infection requires the recognition of HIV-1 CA by the C-terminal CypA domain of TrimCyp. In addition, the CypA/CA interaction in HIV-1 was shown to correlate with viral sensitivity to rhesus and African green monkey Trim5α restriction [19,26,27]. Blocking CypA/CA interaction by CsA or down-regulating endogenous CypA by RNAi rescues HIV-1 replication from rhesus and African green monkey Trim5α restriction, suggesting the rhesus and African green monkey Trim5α restrict HIV-1 via the CypA-binding pathway. Human Trim5α, on the other hand, weakly restricts HIV-1 via a pathway that is independent of CypA binding [26-28]. It is not clear whether the Trim5α restriction is mediated by direct binding to CypA or requires an unidentified adaptor protein. Another explanation is that CypA/CA interaction changes the CA conformation which provides access for rhesus and African green monkey but not human Trim5α. We report here that SIVagmTAN interacts with CypA, but it has a different recognition pattern by different Trim5α proteins when compared to HIV-1 [29]. SIVagmTAN is resistant to the African green monkey Trim5α but susceptible to rhesus Trim5α restriction, whereas HIV-1 is restricted by rhesus and African green monkey Trim5α via the CypA-binding pathway. The SIVagmTAN susceptibility to rhesus Trim5α is probably determined by the patch on the B30.2 domain and the CypA/CA has little to do with the restriction [30].

CypA binding is necessary for Trim5α restriction of HIV-1 in rhesus cells, but not for that in human cells. It has been hypothesized that CypA binding protects HIV from an unknown restriction factor in humans. If so, then this hypothesis would have to be extended to account for the fact that diverse lentiviruses also bind CypA. Our report here will provide a parallel line to investigate the role that CypA plays in lentiviral life cycle.

## Methods

### Cells

293T and CRFK (Crandall Feline Kidney) cells were cultured in Dulbecco's modified Eagle medium with 10% fetal bovine serum (FBS). Jurkat T cells were grown in RPMI with 10% FBS. 293T cells were used for generation of vesicular stomatitis virus G protein (VSV-G) pseudotyped lentiviruses and for production of GST-CypA fusion proteins. CRFK cells expressing TrimCyp protein from different species were generated as described previously [30].

### Generation of VSV-G pseudotyped lentiviruses

2.5 × 10^5 ^cells/ml of 293T cells were plated in 2 ml/well in a 6-well plate 16 hours prior to transfection. The VSV-G pseudotyped HIV-1 WT-GFP, HIV-1 G89V-GFP mutant, HIV-1 WT-Luc, SIVmac-Luc, and SIVagmTAN-Luc were generated as described previously [30,31]. Plasimds pFGinSin and pFP93 [32] used to generate FIV vectors and virions were gifts of Eric Poeschla (The Mayo Clinic College of Medicine, Rochester). For generating VSV-G pseudotyped FIV WT-GFP, 0.75 μg of enhanced green fluorescent protein transfer vector pFGinSin was co-transfected with 0.4 μg of pL-VSV-G, 0.1 μg of pCMV-tat, and 0.75 μg of pFP93 to 293T cells by FuGene 6 transient transfection. For generating the VSV-G pseudotyped mutant FIVs, the same method was applied but replacing pFP93 with pFP93 proviral DNA with mutations of P84A, P85A, P88A, P90A, P92A, and R89A. The mutations were generated with the QuickChange Site-directed mutagenesis kit by following the instruction of manufacturer's protocol (Stratagene). Culture media were collected on 48 hours, 72 hours, and 96 hours after transfection, and were passed through a 0.2 μm filter (Nalgene) for harvesting the viruses. FIV P84A-GFP and FIV P90A-GFP were ultra-centrifuged in an SW28 rotor at 23000 rpm for 1.5 hours for concentrating viruses 10-fold and 100-fold, respectively. All viruses were aliquoted in 1.5 ml micro-tubes and frozen at -80°C until use. The FIV viral titer was tested by infecting CRFK cells with different volumes of the original viral stocks; the amount of virus causing 20% GFP+ (20% infected cells) was defined as 0.2 CRFK infectious equivalents.

### Infection

CRFK or CRFK cells expressing TrimCyp proteins were plated 16 hours before infection at a density of 8 × 10^4 ^cells/ml on a 12-well (1 ml/well) or a 24-well (0.5 ml/well) plate. Jurkat T cells for the CypA-dependency experiment were prepared before infection at a density of 2.5 × 10^5 ^cells/ml on a 12-well (1 ml/well) plate. The VSV-G pseudotyped viruses were added to target cells in the presence of 20 μg/ml DEAE/Dextran, and spinoculated at 1200 relative centrifugal force (rcf) at room temperature for 2 hours [33]. The infected cells were placed at 37°C incubator for 48 hours until the analysis of the infectivity tested with flow cytometry (for viruses expressing GFP) or luminometer (for viruses expressing luciferase). The HIV-1 WT-GFP, HIV-1 G89V-GFP, and all FIVs (WT and mutants) infected cells was harvested and fixed with 1% paraformaldehyde for 1 hour at 4°C. The fixed cells were washed with PBS and re-suspended with 300 μl of PBS supplemented with 5% calf serum, and subjected to FACScan (Becton Dickinson) for the analysis of the infectivity. The HIV-1 WT-Luc, SIVmac-Luc, and SIVagmTAN-Luc infected cells were washed with PBS and lyzed with 80 μl of the cell culture lysis buffer (Promega), and 10 μl of the lysates were used for the measurement of luciferase activity (infectivity) with the Luciferase assay system (Promega) and luminometer.

### GST-CypA pull down assay

The mRNA from 293T cells were extracted with RNeasy kit (Qiagen). The human CypA was cloned from the human mRNA by using the OneStep RT-PCR (Qiagen) with primer sets 5'-GGATCCACGGTTCAGGTGGTTCTGGAGGTTCAGGAGTCAACCCCACCGTGTTC-3' (forward) and 5'-CTCGAGTTATTCGAGTTGTCCACAGTCAGCAATGGT-3' (reverse). The cyclophilin cDNA was used to replace huTrim5α in the plasmid pEF/GST-huTrim5α [34] (a kind gift of Jeremy Luban, Columbia University, New York). To generate GST-CypA protein in human cells, 1 μg of pEF/GST-CypA or pEF/GST were transiently transfected to 293T cells with FuGene 6 (Roche). 72 hours after transfection, one 6-well plate of cells were harvested and lysed with 1.6 ml of GST buffer (50 mM Tris pH 8.0, 150 mM NaCl, 0.5% NP40, 0.1% SDS, and supplemented with complete protease inhibitor). The lysates were centrifuged at 13200 rpm for 1 minute, and the supernatants were harvested. To balance the glutathione-sepharose beads (BD Biosciences) for optimal binding, 200 μl of beads were centrifuged at 5000 rpm for 5 minutes and washed with 800 μl of GST buffer for 3 times. The beads were re-suspended with 200 μl of GST buffer and incubated with 800 μl of GST or GST-CypA supernatants at 4°C on a slowly rotated shaker for 1 hour. After incubation, beads were centrifuged at 5000 rpm for 5 minutes and washed three times with 800 μl of GST buffer. Viruses for the pull down assay (HIV-1 WT, FIV WT, and FIV mutants) were prepared by concentrating the same amount of virions (about 1 ml) into 200 μl of PBS. The concentrated virions then were incubated with the GST or GST-CypA bound beads at 4°C on a slowly rotated shaker for 1 hour. Beads were centrifuged and washed with 800 μl of GST buffer for 3 times. The beads then were re-suspended with 60 μl of GST buffer and frozen at -80°C until the Western blot analysis.

### Western blot analysis

Lysates from the GST-pull down assay were mixed with 20 μl of sample buffer (2% SDS, 1% 2-mercaptomethanol, 1% glycerol, 65 mM Tris-hydrochloride [34]). 30 μl of the mixture were loaded on SDS-10% polyacrylamide gels. After electrophoresis, the proteins were transferred to polyvinylidene difluoride membranes. The membranes were blocked for 30 minutes at room temperature with 5% fat-free milk in PBS, and then incubated with a 1:1000 dilution of a mouse anti-FIV capsid monoclonal antibody (clone 2C1, from NIH AIDS Research and Reference Reagent program) overnight at 4°C. The membranes were washed 10 minutes for 3 times in PBS containing 0.1% Tween 20 and then incubated with a 1:10000 dilution of a horseradish peroxidase-conjugated anti-mouse monoclonal antibody (Santa Cruz Biotechnology) for 30 minutes at room temperature. The membranes were washed 3 times for 30 min, and the bound antibody was detected with the ECL detection system (Amersham).

### Sequence alignment and structure computer modeling

Lentiviral Gag sequences were from GenBank. The accession numbers are: HIV-1 (M19921), HIV-2 (X05291), SIVcpz (AF115393), SIVmac (AY588946), SIVsm (AF334679), SIVagmTAN (U58991), and FIV (M25381). The CA sequences from these lentiviruses were aligned by the ClustalX software [35,36]. The computer modeling of N-terminal CA structure from SIVmac and SIVagmTAN was performed with the SWISS-MODEL server [37, 38, 39] by using the CA crystal structure of HIV-1 which bound to human CypA (PDB:1AK4) [4]. The predicted CA structures were viewed and displayed by DeepView (Swiss Pdb-Viewer).

## Competing interests

The author(s) declare that they have no competing interests.

## Authors' contributions

TYL and ME designed the experiments. TYL performed the experiments. TYL and ME analyzed the data and wrote the paper.
